# Allosteric Communication in the Multifunctional and Redox NQO1 Protein Studied by Cavity-Making Mutations

**DOI:** 10.3390/antiox11061110

**Published:** 2022-06-02

**Authors:** Juan Luis Pacheco-Garcia, Dmitry S. Loginov, Ernesto Anoz-Carbonell, Pavla Vankova, Rogelio Palomino-Morales, Eduardo Salido, Petr Man, Milagros Medina, Athi N. Naganathan, Angel L. Pey

**Affiliations:** 1 Departamento de Química Física, Universidad de Granada, Av. Fuentenueva s/n, 18071 Granada, Spain; 2 Institute of Microbiology—BioCeV, Academy of Sciences of the Czech Republic, Prumyslova 595, 252 50 Vestec, Czech Republic; dmitry.loginov@biomed.cas.cz (D.S.L.); pman@biomed.cas.cz (P.M.); 3 Departamento de Bioquímica y Biología Molecular y Celular, Facultad de Ciencias, Instituto de Biocomputación y Física de Sistemas Complejos (BIFI) (GBsC-CSIC Joint Unit), Universidad de Zaragoza, 50009 Zaragoza, Spain; eanoz@unizar.es (E.A.-C.); mmedina@unizar.es (M.M.); 4 Institute of Biotechnology—BioCeV, Academy of Sciences of the Czech Republic, Prumyslova 595, 252 50 Vestec, Czech Republic; pavla.vankova@ibt.cas.cz; 5 Department of Biochemistry, Faculty of Science, Charles University, Hlavova 2030/8, 128 43 Prague, Czech Republic; 6 Departamento de Bioquímica y Biología Molecular I, Facultad de Ciencias y Centro de Investigaciones Biomédicas (CIBM), Universidad de Granada, 18016 Granada, Spain; rpm@ugr.es; 7 Center for Rare Diseases (CIBERER), Hospital Universitario de Canarias, Universidad de la Laguna, 38320 Tenerife, Spain; edsalido@gmail.com; 8 Department of Biotechnology, Bhupat & Jyoti Mehta School of Biosciences, Indian Institute of Technology Madras (IITM), Chennai 600036, India; athi@ijtm.ac.in; 9 Departamento de Química Física, Unidad de Excelencia en Química Aplicada a Biomedicina y Medioambiente e Instituto de Biotecnología, Universidad de Granada, Av. Fuentenueva s/n, 18071 Granada, Spain

**Keywords:** antioxidant defense, flavoprotein, FAD binding, structural perturbation, protein core, allosterism, cavity-making mutation

## Abstract

Allosterism is a common phenomenon in protein biochemistry that allows rapid regulation of protein stability; dynamics and function. However, the mechanisms by which allosterism occurs (by mutations or post-translational modifications (PTMs)) may be complex, particularly due to long-range propagation of the perturbation across protein structures. In this work, we have investigated allosteric communication in the multifunctional, cancer-related and antioxidant protein NQO1 by mutating several fully buried leucine residues (L7, L10 and L30) to smaller residues (V, A and G) at sites in the N-terminal domain. In almost all cases, mutated residues were not close to the FAD or the active site. Mutations L→G strongly compromised conformational stability and solubility, and L30A and L30V also notably decreased solubility. The mutation L10A, closer to the FAD binding site, severely decreased FAD binding affinity (≈20 fold vs. WT) through long-range and context-dependent effects. Using a combination of experimental and computational analyses, we show that most of the effects are found in the apo state of the protein, in contrast to other common polymorphisms and PTMs previously characterized in NQO1. The integrated study presented here is a first step towards a detailed structural–functional mapping of the mutational landscape of NQO1, a multifunctional and redox signaling protein of high biomedical relevance.

## 1. Introduction

Protein residues are coupled to each other by hydrogen-bond networks and packing interactions causing correlated motions and fluctuations in equilibrium that are critical for protein function [1]. These networks enable the *transmission* of signals due to mutations or PTMs, playing critical roles in multiple protein functions through complex mechanisms [1,2]. Since mutational effects can propagate to long distances [1,2,3,4] and many flavoproteins are multifunctional [2], our understanding of the mutational effects in structure–function relationships is limited by detailed experimental and computational analysis. Generally, the transmission of these mutational effects to distant functional effects can be referred to as allosteric communication (the basis of allosterism). 

In this work, we carried out a detailed biochemical, biophysical, structural and computational analysis of the NAD(P)H quinone:oxidoreductase 1 enzyme (NQO1), a flavoprotein that displays multiple functions (reduction of quinones, xenobiotic detoxification, superoxide scavenging, and interaction with other macrobiomolecules) and shows allosteric communication of mutational and ligand binding effects to different functional sites [5,6,7,8]. NQO1 is a homodimeric flavoprotein that contains a tightly bound FAD molecule per subunit, and it is involved in the two-electron reduction of substrates, including cancer prodrugs, vitamins and superoxide radicals [5,9]. Additionally, NQO1 is associated, due to polymorphisms, mutations and altered expression levels, with several diseases such as cancer, Alzheimer’s disease and Parkinson’s disease [5,9]. Structurally, the protein is divided into two different domains: a large N-terminal domain (NTD; approximately residues 1–225) that harbors most of the active site residues and a tightly bound FAD molecule and a C-terminal domain (CTD; approximately residues 225–274) that completes the active site (i.e., the NAD(P)H and substrate binding sites) and the monomer:monomer interface [10,11,12,13,14]. The NQO1 catalytic cycle follows a ping-pong mechanism: in the reductive half reaction, a NAD(P)H molecule binds to the active site and reduces FAD to FADH_2_; in the oxidative half reaction, the substrate binds and it is reduced by FADH_2_, regenerating the FAD in the oxidized state. Remarkably, the two active sites (one per protomer, with FAD bound and the NADH/NADPH and substrate binding sites) are non-equivalent: both the oxidative and reductive half reactions in the first active site are one order of magnitude faster than the same half reaction in the second active site, thus explaining the negative cooperativity observed in steady-state experiments [7,15]

**Figure 1:**
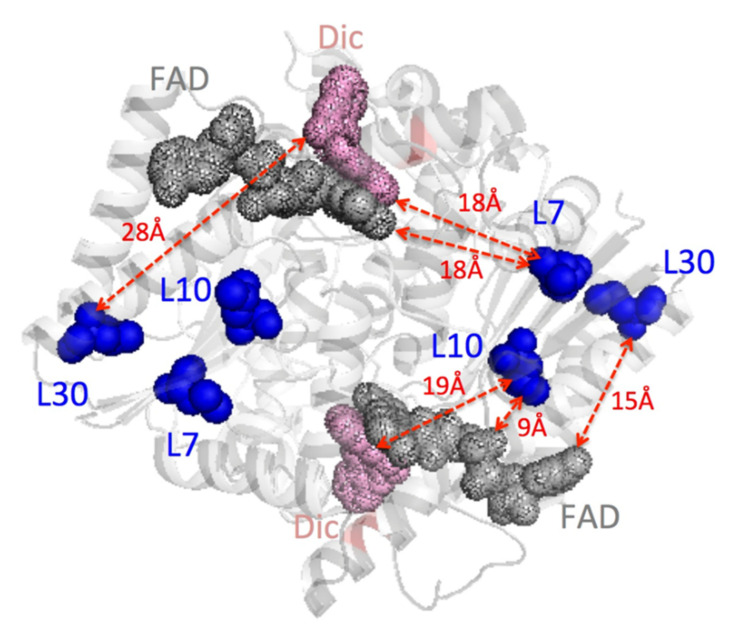
**Structural location of the leucine residues mutated in this work.** Leucine residues are indicated in blue. FAD is indicated in grey and Dic in pink. The figure was generated using the structure with PDB code 2F1O [25]. Solvent-accessible surface areas for L7, L10 and L30 are 0.3 ± 0.2%, 0.4 ± 0.2% and strictly 0%, respectively, using GetArea (http://curie.utmb.edu/getarea.html: accessed on 1 October 2019). Shortest distances between Leu residues and FAD and Dic molecules are indicated with red dashed lines.

To test the presence and extent of allosteric communication between different functional sites in NQO1, we have generated in this work cavity-making mutations of fully buried Leu residues (at L7, L10 and L30) to Val, Ala and Gly (Figure 1). This approach has been largely used to investigate the contribution of the hydrophobic effect, the role of packing interactions on conformational stability and the propagation of mutational effects on protein structure and function [1,16]. To the best of our knowledge, this approach has never been applied to a human flavoprotein. The Leu residues selected in NQO1 are in most of the cases far from the FAD or NADH/dicoumarol binding pockets in the active site (Figure 1) [dicoumarol (Dic) is represented because it is a competitive inhibitor of NAD(P)H and crystal structures are available]. Our results establish that these leucine residues are in long-range allosteric communication with active site residues in a highly context-dependent manner, leading in some cases to counterintuitive effects on several of the multiple functional features of this flavoenzyme. 

## 2. Materials and Methods

### 2.1. Protein Expression and Purification

Mutations were introduced by site-directed mutagenesis in the wild-type (WT) NQO1 cDNA cloned into the pET-15b vector (pET-15b-NQO1) by GenScript (Leiden, The Netherlands). Codons were optimized for expression in *Escherichia coli* and mutagenesis was confirmed by sequencing the entire cDNA. The plasmids were transformed in *E. coli* BL21(DE3) cells (Agilent Technologies, Santa Clara, CA, USA) for protein expression. These constructs contain a hexa-his N-terminal tag for purification. 

**Figure 2:**
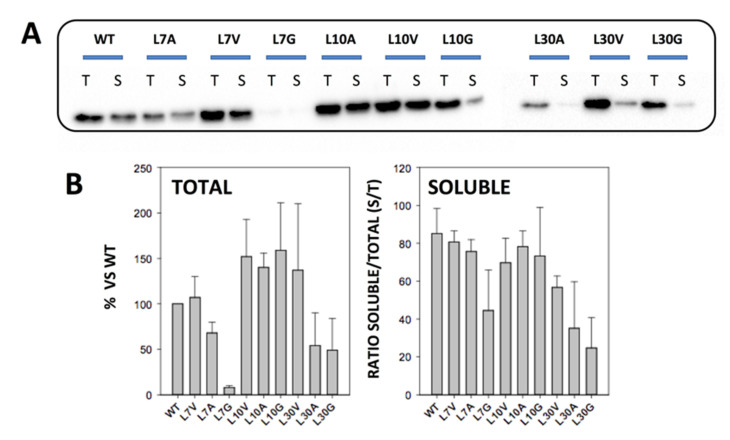
**Expression levels and solubility of NQO1 variants.** (**A**) Representative Western blot analysis of NQO1 total (T) and soluble (S) extracts from *E. coli* cells; (**B**) Quantitative analysis of NQO1 from expression levels (from six independent experiments; mean ± s.d.) solubility is displayed as the ratio of soluble/total protein. Expression was carried out at 37 °C for 4 h upon induction with IPTG 0.5 mM.

To determine the amount of soluble NQO1, 5 mL of LB medium containing 0.1 mg·mL^−1^ ampicillin (LBA) (purchased from Canvax Biotech, Córdoba, Spain) was inoculated with transformed cells and grown for 16 h at 37 °C. A volume of 0.5 mL of these cultures was diluted into 10 mL of LB containing 0.1 mg·mL^−1^ ampicillin and grown at 37 °C for 3 h. After that, cultures were induced with 0.5 mM of isopropyl β-D-1-thiogalactopyranoside (IPTG, Canvax Biotech, Córdoba, Spain) at 37 °C for 4 h. Cells were harvested by centrifugation at 2900× *g* in a bench centrifuge at 4 °C and frozen at −80 °C for 16 h. Cells were resuspended in binding buffer (20 mM Na-phosphate, 300 mM NaCl, 50 mM imidazole, pH 7.4) with 1 mM phenylmethylsulfonyl fluoride (PMSF, Sigma-Aldrich, Madrid, Spain) and sonicated in an ice bath. A volume of 1 mL were taken as *total extracts* and 1 mL was centrifugated (24,000× *g*, 30 min, 4 °C in a bench centrifuge) to obtain the *soluble extracts* (Figure 2). The amount of NQO1 in total and soluble extracts was determined by Western blot analysis (Figure 2). Samples were denatured using Laemmli’s buffer and resolved in 12% acrylamide SDS-PAGE gels and transferred to PVDF membranes (GE Healthcare, Chicago, IL, USA) using standard procedures. Immunoblotting was carried out using primary monoclonal antibody against NQO1 (sc-393736, Santa Cruz Biotechnology, Dallas, TX, USA) at 1:500 dilution and, as secondary antibody, we used an anti-mouse IgGκ BP-HRP (sc-516102, Santa Cruz Biotechnology) at 1:2000 dilution. Samples were visualized using Luminol-based enhanced chemiluminescence (from BioRad Laboratories, Hercules, CA, USA), scanned (using a Chemidoc XRS+ system from BioRad Laboratories, Hercules, CA, USA) and analyzed using Image Lab (from BioRad Laboratories). 

For large-scale purifications, a preculture (40 mL) was prepared from a single clone for each variant and grown for 16 h at 37 °C in LBA and diluted into 2.4–4.8 L. After 3 h with shaking (200 rpm) at 37 °C, NQO1 expression was induced by the addition of 0.5 mM IPTG for 6 h at 25 °C. Cells were harvested by centrifugation at 8000× *g* and frozen overnight at −80 °C. NQO1 proteins were purified using immobilized nickel affinity chromatography columns (IMAC, GE Healthcare) and size-exclusion chromatography (SEC) as described [15] (Figure S1). Isolated dimeric fractions of NQO1 variants were exchanged to HEPES-KOH buffer 50 mM pH 7.4 using PD-10 columns (GE Healthcare). The UV–visible spectra of purified NQO1 proteins were registered in a Cary spectrophotometer (Agilent Technologies, Waldbronn, Germany) and used to quantify the content of FAD as described in [15]. For the samples for pre-steady state kinetic analyses, NQO1 proteins were incubated with 1 mM FAD and excess FAD was removed using PD-10 columns, obtaining a saturation fraction (FAD:NQO1 monomer) higher than 90% based on UV–visible spectra. Apo proteins were obtained by treatment with 2 M urea and 2 M KBr as described [6], obtaining samples with less than 2% saturation fraction of FAD based on UV–visible spectra. Samples were stored at −80 °C upon flash freezing in liquid N_2_. Protein purity and integrity were checked by polyacrylamide gel electrophoresis in the presence of sodium dodecylsulphate (SDS-PAGE) (Figure S1). 

### 2.2. Thermal Stability

Thermal denaturation of NQO1 proteins, as holo proteins (2 μM in monomer + 100 μM FAD), was monitored by following changes in tryptophan emission fluorescence in HEPES-KOH 50 mM at pH 7.4 as described in [17]. T_m_ values are indicated as the mean ± s.d. of four replicates.

### 2.3. Partial Proteolysis by Thermolysin

NQO1 samples (10 μM in monomer) were prepared in HEPES-KOH 50 mM at pH 7.4 in the presence of 100 μM FAD (NQO1_holo_) and 100 μM FAD + 100 μM Dic (NQO1_dic_) in a volume of 135 μL and incubated at 25 °C for 5 min. Thermolysin (from *Geobacillus stearothermophilus*, Sigma-Aldrich, St. Louis, MO, USA) was prepared at 1 and 5 μM (protease concentration) in HEPES-KOH 50 mM at pH 7.4 and 100 mM CaCl_2_. To trigger the reaction, solutions of thermolysin were added to those of NQO1 (previously preincubated at 25 °C for 5 min) at a 1:10 ratio. Samples were collected over time and the reaction quenched by adding EDTA pH 8 (final concentration of 20 mM) and Laemmli’s buffer (2×). Controls (time 0) were prepared likewise but with no added thermolysin. Experiments were carried out at 25 °C. Samples were resolved by SDS-PAGE under reducing conditions in gels containing 12% acrylamide as resolving gel and 4% acrylamide as stacking gel. Gels were stained with Coomassie blue G-250. Densitometry was carried out using ImageJ. Proteolysis reactions were analyzed using an exponential function to provide the apparent rate constant (*k*_obs_). 

### 2.4. FAD Content

FAD content was determined spectroscopically as described in [18]. Briefly, the UV–visible spectra of purified NQO1 proteins were registered in a Cary 100 spectrophotometer (Agilent). The UV–visible spectra were normalized considering that the UV–visible spectra can be deconvoluted using a ε_280_ = 47,900 M^−1^·cm^−1^ for the apo-NQO1 and ε_280_ = 22,000 M^−1^·cm^−1^ and ε_450_ = 11,300 M^−1^·cm^−1^ for free FAD as previously described [17].

### 2.5. FAD Binding Affinity

Fluorescence titrations were carried out at 25 °C using 1 × 0.3 cm path-length cuvettes in a Cary Eclipse spectrofluorimeter (Agilent Technologies, Waldbronn, Germany). Experiments were carried out in 20 mM K-phosphate, pH 7.4, essentially as described in [19]. Briefly, 20 μL of a 12.5 μM NQO1 stock solution (in subunit) was mixed with 0–500 μL of FAD 10 μM and the corresponding volume of buffer was added to yield a 1 mL final volume. Samples were incubated at 25 °C in the dark for at least 10 min before measurements. Spectra were acquired in the 340–360 nm range upon excitation at 280 nm (slits 5 nm), and spectra were averaged over 10 scans registered at a scan rate of 200 nm·min^−1^. FAD binding fluorescence intensities at 350 nm were fitted using single and identical types of binding sites as described in [19]. 

### 2.6. Enzyme Kinetics for the Reductive Half Reaction with NADH

Fast hydride- and deuteride-transfer reactions (HT and DT, respectively) were carried out under anaerobic conditions using a stopped-flow spectrophotometer as described [15]. Briefly, the reductive half reaction was measured by mixing NQO1_holo_ variants (7.5 µM) with NADH ranging from 7.5 to 100 µM (these refer to final concentrations). Reactions were performed in 20 mM HEPES-KOH, pH 7.4. Data were collected using either NADH or NADD, but using only one of these reducing species in a given experiment. Multiple-wavelength absorption data in the flavin absorption region were collected and processed as described [15]. Time-dependent spectral deconvolution was performed by global fitting analysis and numerical integration using previously described procedures [15]. This deconvolution procedure was carried out considering sequential and irreversible steps in the context of a two-step mechanism (A⟶B⟶C) and was used to determine observed rate constants (*k*_obs_) for these steps as well as the spectroscopic properties of these species (A, B and C). According to our recent study, catalytically relevant NQO1 processes involved steps A⟶B⟶C [15]. Despite practical limitations preventing these measurements reaching pseudo-first-order conditions (such as minimum amount of protein sample for detection of flavin reduction and processes over 75 µM NADH becoming too close to the instrumental death time), hyperbolic dependences of *k*_obs_ vs. NADH concentrations were fitted using Equation (1):

(1) $$k_{obs}=\frac{k_{HT}\cdot \left[NADH\right]}{K_{d}^{NADH}+\left[NADH\right]}$$

where *k*_HT_ is the limiting rate constant for HT and *K*_d_^NADH^ is the apparent equilibrium dissociation constant to a given active site. 

To determine primary kinetic isotopic effects (KIEs) in the HT process [20], the *k*_obs_ for HT and DT was determined by mixing NADH/D with NQO1_holo_ using equimolar concentrations of NQO1_holo_ and NADH or [4*R*-^2^H]-NADD (7.5 µM of each component again experiments at higher NADH concentrations where limited but reactions becoming too fast for detection upon increasing temperature). These apparent KIEs were determined as the ratio of *k*_obs_ values using NADH and NADD. Experiments were carried out at temperatures ranging 6–20 °C. Activation parameters (frequency factor, A, and the activation energy, E_a_) were determined using the Arrhenius equation as described [15].

### 2.7. Hydrogen/Deuterium Exchange Mass Spectrometry (HDXMS) 

The structural impact of cavity-making mutations (L7V, L7A, L10V, L10A, L30V, and L30A) in NQO1 was evaluated using hydrogen/deuterium exchange (HDX) coupled to mass spectrometry. Proteins were monitored in their NQO1_apo_, NQO1_holo_ and NQO1_dic_ states with the only exception of L30A mutant being analyzed solely as NQO1_holo_ and NQO1_dic_. To start the HDX reaction, 20 µM protein was diluted 10 fold into a D_2_O-based buffer [50 mM HEPES-KOH, 0.5 mM TCEP (tris(2-carboxyethyl)phosphine hydrochloride, Sigma-Aldrich, St. Louis, MO, USA), pD 7.4]. Under NQO1_holo_ and NQO1_dic_ conditions, 20 µM protein variants were pre-incubated at least for 10 min with 200 µM FAD (NQO1_holo_) or with 200 µM FAD + 200 µM dicoumarol (NQO1_dic_). After 10, 50, 250, 1250 and 6250 s of HDX the reaction was quenched by mixing with 0.5 M Glycine-HCl, pH 2.3 in ratio 1:1 and samples were frozen in liquid nitrogen. The time points 10, 250 and 6250 s were prepared as duplicates. Subsequently, each sample was thawed and injected onto the LC system including serially coupled immobilized protease columns (nepenthesin-2 and pepsin) where it was digested for 3 min by flow of 0.4% formic acid (FA) in water, delivered at a flow rate of 200 µL min^−1^ (1260 Infinity II Quaternary pump, Agilent Technologies, Waldbronn, Germany). Generated peptides were online trapped and desalted on SecurityGuard™ pre-column (ULTRA Cartridges UHPLC Fully Porous Polar C18, 2.1 mm, Phenomenex, Torrance, CA, USA). Next, peptides were separated on a reversed-phase analytical column (LUNA^®^ Omega Polar C18 Column, 100 Å, 1.6 µm, 100 mm × 1.0 mm, Phenomenex, Torrance, CA, USA) at a flow rate of 40 µL min^−1^ using a 10–40% linear gradient of solvent B (A: 0.2% acetonitrile/0.1% FA in water; B: 98% acetonitrile/0.1% FA in water) (1290 Infinity II LC system, Agilent Technologies, Waldbronn, Germany) as described [6]. Digestion and separation were performed at 0 °C and pH 2.3 to minimize deuterium loss. The LC system was directly interfaced with an ESI source of 15T FT-ICR mass spectrometer (SolariX XR, Bruker Daltonics, Bremen, Germany). Data were exported and processed using the Data Analysis v. 5.3 (Bruker Daltonics, Bremen, Germany) and in-house developed DeutEx software [21]. Peptide identification for each variant was performed by data-dependent LC–MS/MS measurement using the same LC system and gradient elution but with ESI-timsTOF Pro PASEF instrument (Bruker Daltonics, Bremen, Germany). Peptides were identified by a MASCOT (Matrix Science, London, UK) search against a custom-built database combining sequences of NQO1 variants and contaminants from the cRAP database. Decoy search was enabled, the false discovery ratio was set to 1% and the ion score cut-off to 20. Exchange rates were corrected for back exchange as described previously [6,22]. To evaluate the effect of mutations, the difference in kinetics of deuterium incorporation (% D vs. time) of mutants and the WT protein was calculated for a given ligation state and for each protein segment experimentally determined, and the average of the two most different time points (mutant-WT) was determined (i.e., Δ%D_av_). This procedure enabled determining the difference in HDX kinetics between two given NQO1 states as recently described [2,6].

### 2.8. Statistical Mechanical Model Predictions 

The block version of the Wako–Saitô–Muñoz–Eaton model (bWSME) was employed by fixing the parameters to those from a recent analysis of NQO1 folding thermodynamics [23]. A detailed description of the model and model parameterization has been provided in a recent work [24]. Briefly, the residue-level coarse graining in the conventional Gō- and Ising-like WSME model is extended to included stretches of consecutive residues or blocks (b, with the most probable block size of 3). The model includes contributions from microstates with single stretches of folded residues (single sequence approximation, SSA), two stretches of folded residues (double sequence approximation, DSA) and DSA, allowing for interactions across the folded islands if they interact in the native structure (DSAw/L). The structure of NQO1 (PDB code 2F1O) [25] is employed to calculate the number and nature of van der Waals interactions (with a 5 Å heavy-atom cut-off), charge–charge interactions (protonation state of pH 7.0) and simplified solvation free energies for every microstate that numbers to 5,002,535, at 310 K and 100 mM ionic strength. The statistical weights of each of these microstates are summed up to calculate the overall partition function and partial partition functions, from which the free energy profiles are predicted. 

Once the probabilities of every microstate are calculated, the ensemble is split into two sub-ensembles, which account for the probability of residue *i* to be folded with respect to the folding probability of residue *j*, to calculate coupling free energies [26]. Specifically, $\sum p_{i_{f}j_{f}}$ sums over all states in which both residue *i* and residue *j* are folded while $\sum p_{i_{u}j_{f}}$ accounts for all states in which residue *i* is unfolded and *j* is folded. Following this, the positive coupling free energy is calculated for every residue as:

(2) $$\Delta G_{+}=RTln\left(K_{+}\right)=RTln\left(\frac{\sum p_{i_{f}j_{f}}}{\sum p_{i_{u}j_{f}}}\right)$$

The resulting two-dimensional matrix is termed the positive coupling matrix. The calculation is repeated for the mutant proteins (L7A, L10A, and L30A; mutations introduced via PyMol [27]) with the same set of parameters. 

## 3. Results and Discussion

### 3.1. Expression and Solubility of Cavity-Making Mutants

We mutated three buried Leu residues (L7, L10 and L30) to Val, Ala and Gly (Figure 1). These Leu residues are far from the FAD and Dic molecules, with only L10 being slightly closer than 10 Å to the FAD binding site (Figure 1). Even though codon optimization was used to generate the mutants, we found large differences in total protein and soluble protein levels among them. Western blot analysis of total *E. coli* extracts revealed decreased total protein levels in L7A, L30A and L30G mutants, and particularly for L7G that essentially abolished expression (Figure 2). Additionally, the ratio of the soluble-to-total protein amounts (S/T) showed that L7G, L30V, L30A, and L30G reduced the solubility of NQO1 (Figure 2). These results were confirmed by SEC and SDS-PAGE using large-scale purifications (Figure S1). Therefore, mutations to glycine were not further investigated due to extremely low yields and instability. Overall, these results also indicated that the effects of cavity-making mutations on expression levels and solubility largely depend on the location of the mutated site rather than its burial in the structure. 

### 3.2. Thermal Stability of Cavity-Making Mutants

For those mutants that yielded good expression levels, we carried out further biophysical characterization. This set of mutants were analysed, first by thermal denaturation, which in the case of NQO1 is an irreversible process involving unfolding and dissociation of the protein dimer [18]. Therefore, these analyses do not provide thermodynamic information on mutational effects, but allows a qualitative comparison on their kinetic stability [18]. The experiments were carried out as NQO1_holo_ proteins (purified protein + 100 µM FAD). The mutation L10A had the largest effect, decreasing the T_m_ by 8 °C. Mutations L7V, L7A and L10V showed a more modest destabilization (decreasing the T_m_ by 5–6 °C) (Figure S2). Intriguingly, the mutants L30V and L30A, that showed large effects on protein expression/solubility, only decreased the stability by 2–3 °C, suggesting that these mutants affect the expression/solubility more than thermal stability of the NQO1 dimer.

### 3.3. Local Stability of the NTD Investigated by Partial Proteolysis with Thermolysin

Partial proteolysis with thermolysin has been shown to inform on the local stability of the FAD binding site, with a primary cleavage site between S72 and V73 [14]. The proteolysis pattern is conserved across all mutants investigated (Figure S3). Enhanced sensitivity towards this proteolytic activity often correlates with reduced FAD binding affinity [2,14]. Thus, we carried out proteolysis experiments at two different concentrations of protease for all variants in the NQO1_holo_ and NQO1_dic_ states (i.e., protein as purified + 100 μM FAD + 100 μM dicoumarol) (Figure S3). Interestingly, none of the mutants cause large effects on proteolytic sensitivity (Figure S4 and Table S1). Therefore, these mutations did not seem to affect largely the local stability of the loop 57–66 close to the FAD binding site. 

### 3.4. FAD Binding Affinity

**Figure 3:**
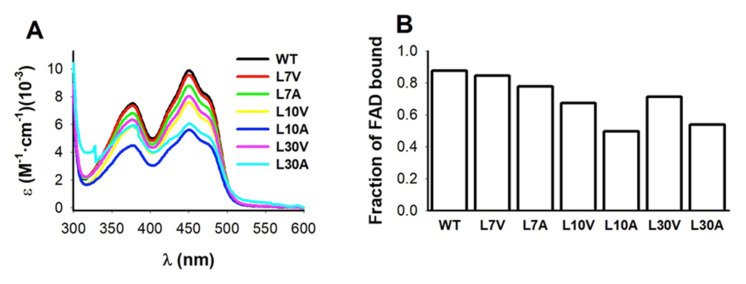
**FAD content of purified NQO1 variants upon purification using IMAC plus SEC.** (**A**) Absorption spectra in the region of absorption of FAD; (**B**) FAD content was calculated using the ratio of absorbance at 280 and 450 nm as described in materials and methods. Data are the average from two different purifications.

**Figure 4:**
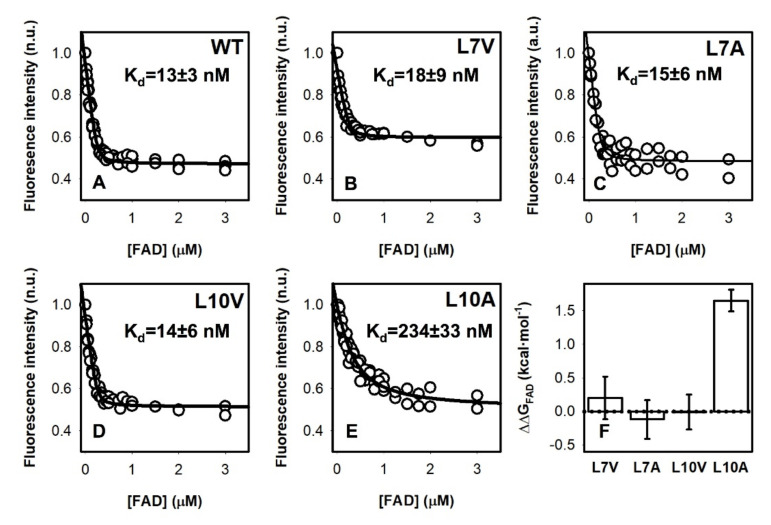
**Fluorescence titrations of NQO1_apo_ variants with FAD.** (**A**–**E**) Experimental data are shown as circles and lines are best fits to a single type of independent binding sites. Data are from at least two independent experiments (N = 4 for WT and N = 2–3 for the mutants). (**F**) The changes in apparent binding free energy including errors from linear propagation. Experiments were performed at 25 °C.

To test potential effects on FAD binding, we first measured FAD content by UV–visible spectroscopy (Figure 3). Three variants (L10V, L10A and L30A) showed lower FAD content than the WT protein. Interestingly, the mutant L30A showed an abnormal spectra, with similar absorption at 375 and 450 nm, suggesting severe effects on the FAD binding mode (Figure 3A). Due to the correlation between FAD content of NQO1 protein variants and their affinity for FAD [14,18,19,28], we carried out direct titrations of apo-proteins with FAD to corroborate potential effects on FAD binding affinity (Figure 4). Mutants at L30 were too unstable to yield enough quantity of pure apo-protein. Nevertheless, we found that the mutant L10A reduced the affinity for FAD by 18 fold compared to the WT protein, which corresponds to a Gibbs free energy penalty of ~1.7 kcal·mol^−1^. This result is also consistent with the expectation based on distance considerations—L10 is the closest to the FAD binding site among the Leu mutants studied (Figure 1).

### 3.5. Effect of Cavity-Making Mutations on Enzyme Kinetics

**Figure 5:**
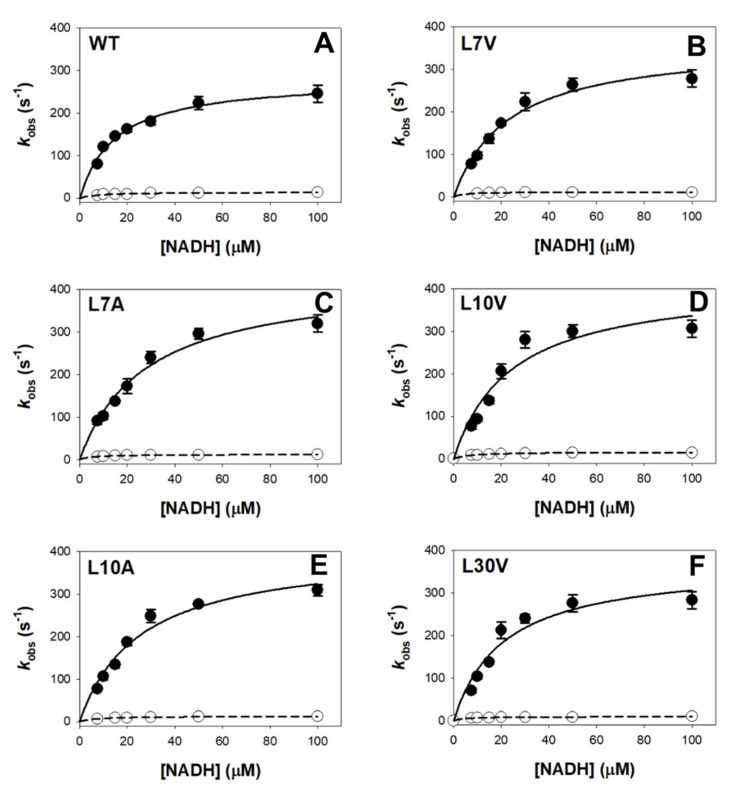
**Dependence of *k*_obs_ on NADH concentration for NQO1 variants.** (**A**–**F**) Enzyme kinetics for different variants (the variant identity is displayed in each panel). Experiments were carried out using 7.5 μM NQO1 at 6 °C. Data are from at least three replicates and displayed as the mean ± s.d. Black circles correspond to the fast pathway, and open circles show data for the slow pathway. Lines are best fits to Equation (1). Each panel shows the data for the given variant.

The reduction kinetics by NAD(P)H of the FAD bound to WT NQO1 is complex [15] (Figure S5). We have observed two different pathways with a difference in apparent rate constants of at least one order of magnitude, supporting the existence of functional negative cooperativity, named as *fast* and *slow* paths [2,15]. The cavity-making mutations had some effects on the catalytic cycle (Figure 5 and Table 1). Mutations L7A and L10A increased the *K*_d_ for NADH by 2 fold in the fast reaction pathway, and the L10A also increased the *K*_d_ for NADH by 3 fold in the slow reaction pathway (Table 1). However, it must be noted that these changes in the *K*_d_ for NADH are often associated with modest increases in the *k*_HT_; therefore, catalytic efficiency is not largely affected in most cases (Table 1).

The influence of mutations on the NQO1 active site dynamics was further studied by evaluating the reductive half reaction with NADH and NADD at different temperatures. We use only those mutants whose solubility extents are not significantly affected, namely L7A, L7V, L10A and L10V. At the low temperature, fast and slow reduction steps were slowed down when using NADD for all variants similarly than in the WT (Table S2), resulting in low KIEs (1.5–2.2). However, some mutants showed temperature dependences of KIEs in contrast to the WT protein (Figure S6). 

The temperature-independent KIEs in WT NQO1 were interpreted (for the fast and slow steps) as transitions under the barrier and tunneling of both proton and deuterium, while KIEs decreasing with temperature may indicate only proton tunnelling [2,15,29]. Analysis of WT data in the context of the Arrhenius equation indicated tunneling ready states with movements of active site heavy atoms do not actively contributing to bring the donor and acceptor to the distance for efficient tunnelling and HT but increase that to achieve tunnel ready conformations (passive dynamics). Replacements at L7 and L10 modulated pre-exponential factors (A) and activation energies (E_a_) in both steps (Figure S6 and Table S2). Larger differences were found for the fast steps in L7V and L7A, and particularly for the slow steps in L10V and L10A (with large changes increases in A values and in activation energies). These changes in activation parameters suggest: (i) Passive dynamics is the major contributor to achieve the proton tunnel ready configuration; (ii) Some reaction steps might require a larger structural reorganization to engage the coenzyme in the catalytic process as well as a little contribution of donor–acceptor distance sampling to achieve tunnel ready conformations. Thus, volume changes at L7 and L10 mildly alter the overall packing and general dynamics of NQO1 active sites, with catalytic enhancement achieved by promoting and optimizing vibrations in active sites that minimize DAD fluctuations [29]. Overall, these observations actually agree well with HDX analyses that showed changes in structural stability in the close environment of the isoalloxazine binding site of NQO1_holo_ and NQO1_dic_ (Section 3.6).

### 3.6. Effect of Cavity-Making Mutations on the Structural Stability of NQO1

To evaluate the effect of cavity-making mutations on the local stability in different ligation states (NQO1_apo_, NQO1_holo_ and NQO1_dic_), we have used hydrogen–deuterium exchange (HDX) followed by mass spectrometry [2,6]

#### 3.6.1. The L7 Cavity-Making Mutants

**Figure 6:**
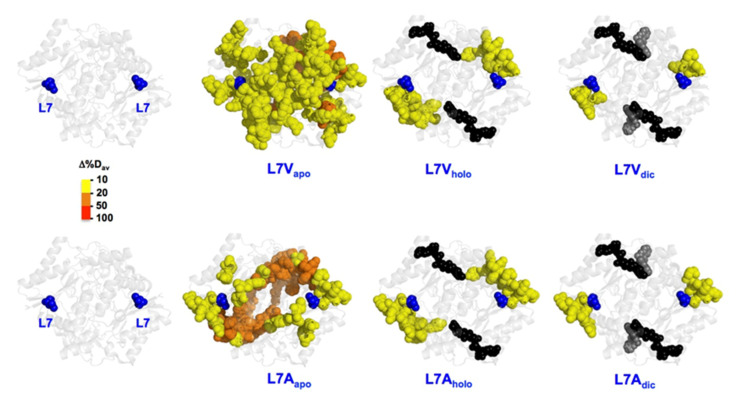
**Representation of the changes in local stability (as Δ%D_av_) due to mutations L7V and L7A vs. WT.** Results are shown as indicated in the color scale for different ligation states (NQO1_apo_, NQO1_holo_ and NQO1_dic_). FAD and Dic are shown as spheres (black and grey, respectively). The structural model used for display was PDB 2F1O [25].

We observed significant changes in the local stability of the NQO1_apo_ state upon mutations L7V and L7A. In the L7V mutant, these effects seemed to spread to longer regions, but were generally mild (Δ%D_av_ of about 10%; in yellow). In the case of L7A, the effects were stronger but more local (Figure 6). Binding of FAD and/or dicoumarol led to a dramatic decrease in these destabilizing effect. Therefore, mutations L7V and L7A seem to mostly target the stability of the NQO1_apo_ state. We must also remember that the L7G mutant almost fully abolished expression of the protein (Figure 2), suggesting that conformational flexibility at this site can be critical for proper expression and/or folding of the protein. Changes in the local stability of the active site (Figure 6) might be associated with the effects observed in enzyme kinetics and dynamics along the HT process. 

#### 3.6.2. The L10 Cavity-Making Mutants

**Figure 7:**
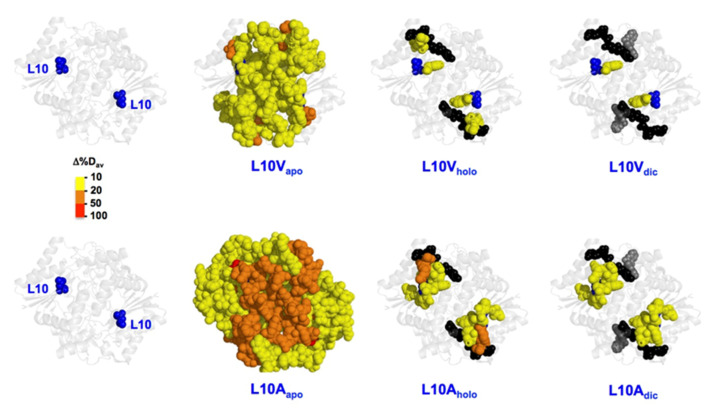
**Representation of the changes in local stability (as Δ%D_av_) due to mutations L10V and L10A vs. WT.** Results are shown as indicated in the color scale for different ligation states (NQO1_apo_, NQO1_holo_ and NQO1_dic_). FAD and Dic are shown as spheres (black and grey, respectively). The structural model used for display was PDB 2F1O [25].

As observed for L7 mutants (Section 3.6.1), the most remarkable changes in local stability caused by mutations at L10 were found in the NQO1_apo_ state (Figure 7). We observed that the propagation of destabilizing effects due to mutations is substantially larger for the L10A than for L10V in the NQO1_apo_ state (Figure 7). However, these effects were largely abolished in the NQO1_holo_ and NQO1_dic_ states, although some residual destabilization was still observed in the L10A mutant in both these states (Figure 7). This large destabilization due to the mutation L10A in the NQO1_apo_ and NQO1_holo_ states could explain the significant decrease for FAD binding affinity experimentally observed (Figure 4). We must also note that the L10G mutation severely reduced the solubility of the protein, making unfeasible its further characterization, and supporting that the combined effect of cavity making plus increased backbone flexibility has dramatic effects on NQO1 foldability. These results revealed a highly specific impact of mutations at L7 and L10 on the apo-state, in contrast to other mutations such as S82D and P187S that had severe impacts in different ligation states [2]. 

#### 3.6.3. The L30 Cavity-Making Mutants

**Figure 8:**
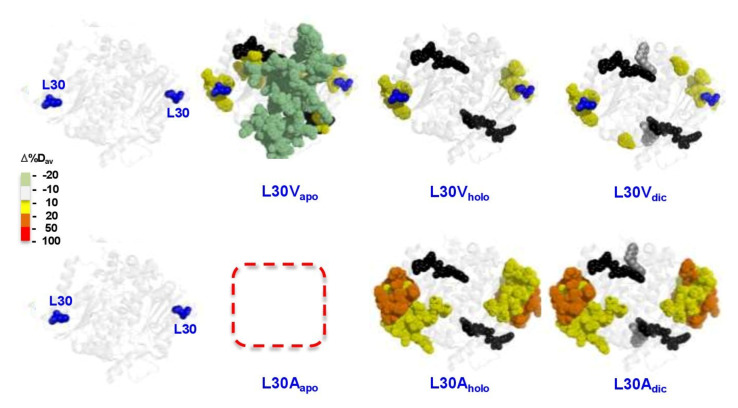
**Representation of the changes in local stability (as Δ%D_av_) due to mutations L30V and L30A vs. WT.** Results are shown as indicated in the color scale for different ligation states (NQO1_apo_, NQO1_holo_ and NQO1_dic_). Note that light green indicates slight stabilization. FAD and Dic are shown as spheres (black and grey, respectively). The structural model used for display was PDB 2F1O [25].

The results obtained by HDXMS for L30V and L30A were striking (the large loss in solubility and stability meant that probing the mutant L30G was not possible in any state) (Figure 2). L30V in the NQO1_apo_ state showed mild destabilization around the mutation, but, surprisingly, other segments of the proteins were mildly stabilized. In the NQO1_holo_ and NQO1_dic_ states, only local and mild destabilization was observed (Figure 8). This could explain why this mutant has little effect on FAD binding, catalytic performance or stability of the protein (Figure 3, Figure 4 and Figure 5, Figures S2 and S3 and Table 1). Still, the mutation L30V reduced the levels of soluble and well folded protein (Figure 2 and Figure S1).

The mutant L30A showed stronger destabilizing effects around the mutated site and these propagated to a longer distance in both NQO1_holo_ and NQO1_dic_ states (Figure 8). For this variant, we could not prepare samples of quality sufficient for HDXMS experiments in NQO1_apo_ state possibly due to solubility/stability issues in this state.

### 3.7. Statistical Mechanical Calculations on the Effects of NQO1 Cavity-Making Mutants

**Figure 9:**
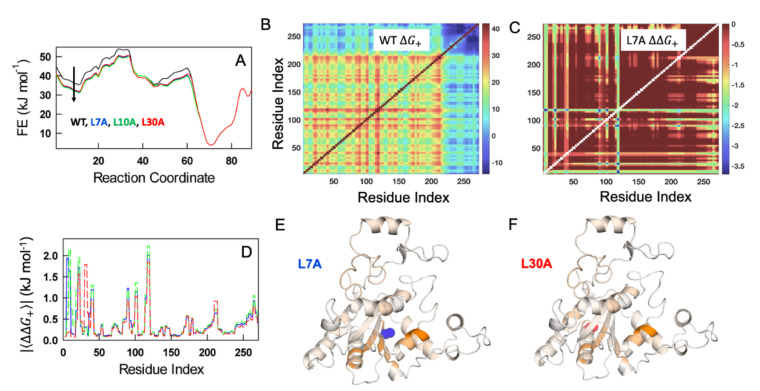
**WSME model predictions on the effect of hydrophobic truncations.** (**A**) Free energy profiles of WT NQO1 (black), L7A (blue), L10A (green), and L30A (red), as a function of the number of structured residues as the reaction coordinate. Note that the free energy of the unfolded state decreases (arrow), highlighting thermodynamic destabilization of the mutants due to the loss of van der Waals interactions upon truncation. (**B**) Positive coupling free energy matrix of the WT. The color bar shown to the right is in kJ mol^−1^. (**C**) Difference in coupling free energy matrices between the WT and the L7A mutant. Color bar is in kJ·mol^−1^. (**D**) Absolute values of the mean differences in positive coupling free energies between the WT and the mutants as a function of residue index. Since the differences are negative (panel (**C**), and as the WT residues are more strongly coupled), absolute values are employed for ease of visualization. (**E**,**F**) The values in panel (**D**) mapped onto the structure for L7A (panel (**E**)) and L30A (panel (**F**)) mutations. Dark orange corresponds to residues that are the most perturbed while white signals little or no change. The structural model used was PDB 2F1O [25].

To explore the role of cavity-making mutations on the conformational landscape of NQO1_apo_, the folding free energy profile of the WT, as a function of the number of structured blocks, at 310 K is predicted employing the bWSME model [26]. The model accounts for an ensemble of more than 5 million microstates or conformational states (see Section 2.8 in Methods) whose probabilities are estimated employing a structure-based algorithm while accounting for van der Waals interactions, electrostatics and solvation free energy terms, apart from residue-level conformational entropy parameters [24]. The resulting free energy profile is indicative of a multistate-like folding behavior with several high free energy intermediates (Figure 9A). Three cavity-creating mutations—L7A, L10A and L30A—were introduced on the WT structure and fed into the model to predict the mutant free energy profiles. The mutant free energy profiles exhibit lower stability (Figure 9A)—note the lower free energy values for the mutants at ~10 structured blocks—as the mutation involves the replacement of a large aliphatic amino-acid (leucine) with a smaller amino acid (alanine). The folded ensemble, at ~70 structured blocks, appears to be relatively unperturbed on mutations. However, this could be a result of the fact that we are lumping together millions of microstates into a specific order parameter, i.e., the number of structured blocks.

The effect on the native ensemble can be more accurately gleaned by estimating the extent to which different residues are coupled to each other in the native ensemble. To this end, we constructed the matrix of positive coupling free energies ($\Delta G_{+}$) that accounts for the sum of probabilities of states in which two residues simultaneously folded vs. those states in which they are decoupled (one residue is folded and the other is not) (Equation (2)). The positive coupling free energy matrix highlights that the majority of the residues are strongly coupled to each other (shades of red in Figure 9B) with the C-terminal region being weakly coupled (shades of blue in Figure 9B). The latter observation is consistent with HDX–MS experiments on WT NQO1_apo_ [2,6]. This calculation is repeated for each of the mutants and a differential coupling matrix ($\Delta \Delta G_{+}=\Delta G_{+,\;mut}-\Delta G_{+,WT}$) is generated to identify protein regions that are perturbed (shown for the L7A mutant in Figure 9C). It is observed that a large number of residues display lower coupling magnitudes in the native ensemble (negative values as the coupling is weaker; colors that are not red in Figure 9C). Plotting the absolute mean values of $\Delta \Delta G_{+}$ averaged across rows (or columns) as function of residue index provides a simpler view of the residue-wise altered coupling pattern (Figure 9D), which can also be visualized by mapping them on to the structure (Figure 9E,F). The average effects are small because we do not consider additional weakening of interactions in the second-shell around the mutated site [1]. However, this calculation suggests that the cavity-creating mutants have stronger effects in the NTD (residues 1–50 and 80–120), and that these uncoupling effects are dependent on the mutated site (Figure 9D–F). To summarize, the bWSME model predicts that mutations modulate the folding probability of multiple structural regions in NQO1 in a non-intuitive manner that is consistent with our experimental characterization carried out on these cavity-making mutants.

## 4. Conclusions

Human flavoproteins display multiple functional features, including oxido-reduction and antioxidant activities, interaction with small effectors and other biomacromolecules and transport to different subcellular and extracellular locations [2]. This multifunctionality is likely imprinted in their energy landscape in which different substates with different energetic and functional features can be populated. Mutations can affect this energy landscape by altering protein interaction networks and leading to allosteric effects due to long-range propagation of mutational effects [1,3,4]. Herein, we have perturbed the interaction network of the human flavoenzyme NQO1 at three buried positions (L7, L10 and L30) in the N-terminal domain by introducing cavity-making mutations (L→V and L→A), which may, additionally, enhance backbone flexibility (L→G). We showed that L→G mutations severely compromised protein folding and/or solubility, and L→V and L→A mutations affected protein stability, flavin binding, catalytic activity and functional cooperativity to different, and not always intuitive, extents. Using a combination of HDX–MS and statistical mechanical calculations, we also showed that these mutations affected the native state ensemble and folding landscape in a site-specific manner, mostly targeting the apo state of the enzyme at L7 and L10. Importantly, our work shows that cavity-making mutations affect the conformational states (e.g., apo-NQO1 and holo-NQO1) differently to naturally occurring and disease-associated mutations [2]. The fact that the pseudo-phosphorylating mutation S82D, the cancer-associated polymorphism P187S as well as natural and artificial mutations at different sites affect both the holo and apo states [2,19,28], whereas cavity making mostly targets the stability of the apo state nicely, shows the multiple pathways available for transmitting mutational effects to different functional features in different ligation states (i.e., allosteric communication). Our approach can be of general application to prove allosteric mechanisms at high resolution for this and other human flavoenzymes, which can in many cases be associated with disease upon mutation (please see the large genetic diversity found just for NQO1 in COSMIC at https://cancer.sanger.ac.uk/cosmic/search?q=NQO1 (accessed date: 20 May 2022) and gnomAD at https://gnomad.broadinstitute.org/gene/ENSG00000181019?dataset=gnomad_r2_1 (accessed date: 20 May 2022).

## Figures and Tables

**Table 1:** Enzyme kinetic analysis for the reductive half reaction of NQO1_holo_ variants with NADH. Primary data are shown in Figure 5.

Variant	*k_HT_*_FAST_ (s^−1^)	*K*_d FAST_ (μM)	*k*_HT_/*K*_d FAST_ (μM^−1^·s^−1^)	*k*_HT SLOW _ (s^−1^)	*K*_d SLOW_ (μM)	*k*_HT_/*K*_d SLOW_ (μM^−1^·s^−1^)
**WT**	281 ± 14	15 ± 2	19 ± 2	14 ± 2	8 ± 3	1.8 ± 0.6
**L7V**	363 ± 26	23 ± 4	16 ± 2	18 ± 1	6 ± 1	3.0 ± 0.5
**L7A**	430 ± 31	28 ± 5	15 ± 2	13 ± 1	5 ± 1	2.5 ± 0.4
**L10V**	417 ± 59	24 ± 8	17 ± 2	16 ± 1	7 ± 1	2.3 ± 0.8
**L10A**	460 ± 30	32 ± 5	14 ± 2	13 ± 1	7 ± 1	1.8 ± 0.1
**L30V**	370 ± 42	21 ± 6	18 ± 2	10 ± 1	5 ± 1	2.1 ± 0.6

## Data Availability

All data are contained within the article and supplementary materials.

## Supplementary Materials

The following supporting information can be downloaded at: https://www.mdpi.com/article/10.3390/antiox11061110/s1. Figure S1: Purification of NQO1 proteins containing mutations at L7, L10 and L30; Figure S2: Thermal stability of NQO1 cavity-making mutants; Figure S3: Representative SDS-PAGE analysis for the proteolytic kinetics of NQO1 variants with thermolysin; Figure S4: Proteolysis kinetics of NQO1 variants with thermolysin; Figure S5: Reductive half reaction of FAD bound to NQO1 variants with NADH; Figure S6: Temperature dependence of kinetic parameters for the two hydride/deuteride transfer (HT/DT) processes from NADH to NQO1. Table S1: Observed rate constants (*k*_obs_) for partial proteolysis of NQO1 variants with thermolysin. Table S2: Arrhenius parameters and KIEs for the HT/DT in the reduction of NQO1 variants by NADH/NADH.
