# Supplementary material for: Allosteric Communication in the Multifunctional and Redox NQO1 Protein Studied by Cavity-Making Mutations
Source: Antioxidants (Basel). 2022 Jun 2;11(6):1110. doi: 10.3390/antiox11061110 (PMC9219786; doi:10.3390/antiox11061110)
Supplement: Supplementary file 1 [file antioxidants-11-01110-s001.zip › antioxidants-1722071-supplementary-done.pdf]

antioxidants

Supplementary Materials: Allosteric Communication in the Multifunctional and Redox NQO1 Protein Studied by Cavity-Making Mutations

Juan Luis Pacheco-Garcia [1,*,†], Dmitry S. Loginov [2,†], Ernesto Anoz-Carbonell [3,†], Pavla Vankova [4,5], Rogelio Palomino-Morales [6], Eduardo Salido [7], Petr Man [2], Milagros Medina [3], Athi N. Naganathan [8] and Angel L. Pey [9,*]

1 Departamento de Química Física, Universidad de Granada, Av. Fuentenueva s/n, 18071 Granada, Spain
2 Institute of Microbiology—BioCeV, Academy of Sciences of the Czech Republic, Prumyslova 595, 252 50 Vestec, Czech Republic; dmitry.loginov@biomed.cas.cz (D.S.L.); pman@biomed.cas.cz (P.M.)
3 Departamento de Bioquímica y Biología Molecular y Celular, Facultad de Ciencias, Instituto de Biocomputación y Física de Sistemas Complejos (BIFI) (GBsC-CSIC Joint Unit), Universidad de Zaragoza, 50009 Zaragoza, Spain; eanoz@unizar.es (E.A.-C.); mmedina@unizar.es (M.M.)
4 Institute of Biotechnology—BioCeV, Academy of Sciences of the Czech Republic, Prumyslova 595, 252 50 Vestec, Czech Republic.; pavla.vankova@ibt.cas.cz or pavla.vankova@biomed.cas.cz
5 Department of Biochemistry, Faculty of Science, Charles University, Hlavova 2030/8, 128 43 Prague, Czech Republic
6 Departamento de Bioquímica y Biología Molecular I, Facultad de Ciencias y Centro de Investigaciones Biomédicas (CIBM), Universidad de Granada, 18016 Granada, Spain; rpm@ugr.es
7 Center for Rare Diseases (CIBERER), Hospital Universitario de Canarias, Universidad de la Laguna, 38320 Tenerife, Spain; edsalido@gmail.com
8 Department of Biotechnology, Bhupat & Jyoti Mehta School of Biosciences, Indian Institute of Technology Madras (IITM), Chennai 600036, India; athi@ijtm.ac.in
9 Departamento de Química Física, Unidad de Excelencia en Química Aplicada a Biomedicina y Medioambiente e Instituto de Biotecnología, Universidad de Granada, Av. Fuentenueva s/n, 18071 Granada, Spain
* Correspondence: juanlupacheco@correo.ugr.es (J.L.P.-G.); angelpey@ugr.es (A.L.P.); Tel.: +34-958243173 (A.L.P.)
† These authors contributed equally to this work.

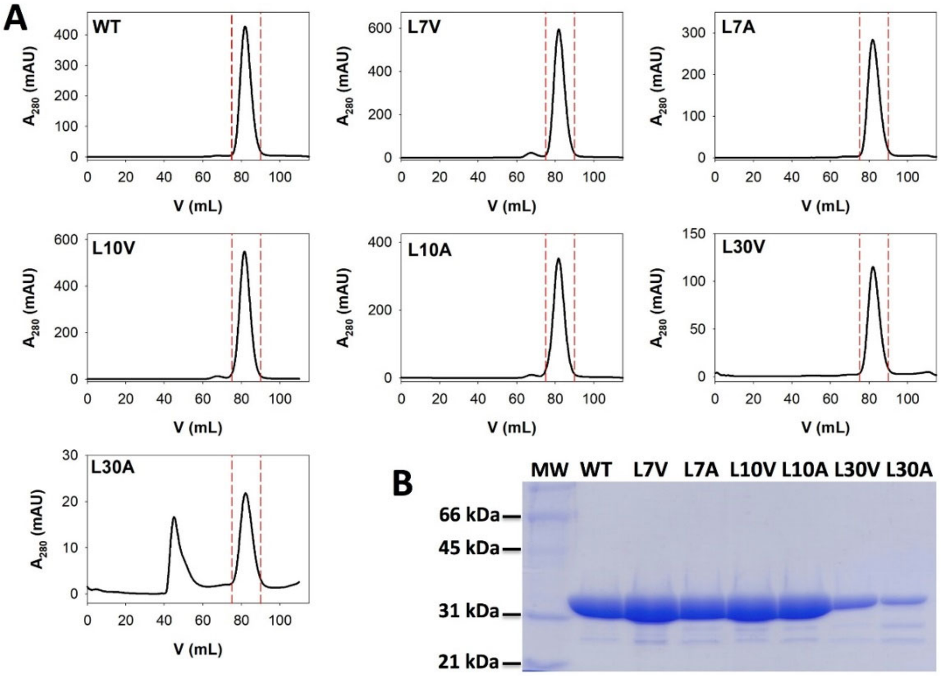

Figure S1. Purification of NQO1 proteins containing mutations at L7, L10 and L30. A) SEC profiles of NQO1 proteins previously isolated by IMAC. Each experiment corresponds to the chromatographic separation of the protein isolated by IMAC from 2.4-4.8 L of bacterial culture.

Chromatography was carried out in a HiLoad® 16/600 Superdex® 200 prep grade column (GE Healthcare) using 20 mM HEPES-KOH 200 mM NaCl at pH 7.4 as mobile phase. Vertical dashed lines correspond to the fractions (volume 75-90 mL) collected and containing NQO1 dimeric fraction. B) SDS-PAGE analyses (12 % acrylamide) of NQO1 dimeric fractions obtained from profiles shown in panel A.

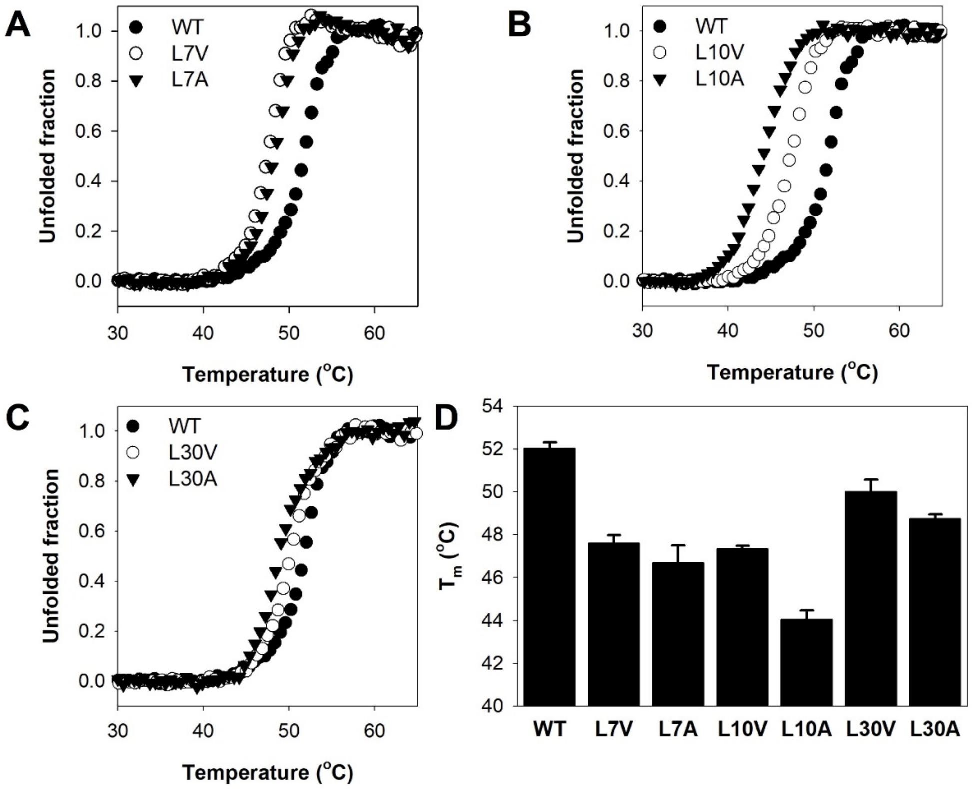

Figure S2. Thermal stability of NQO1 cavity-making mutants. Panels A-C show representative experimental thermal denaturation experiments. Panel D displays the average ± s.d. from at least four different replicates. The experiments were carried out using 2 μM of proteins (in monomer) and 100 μM FAD.

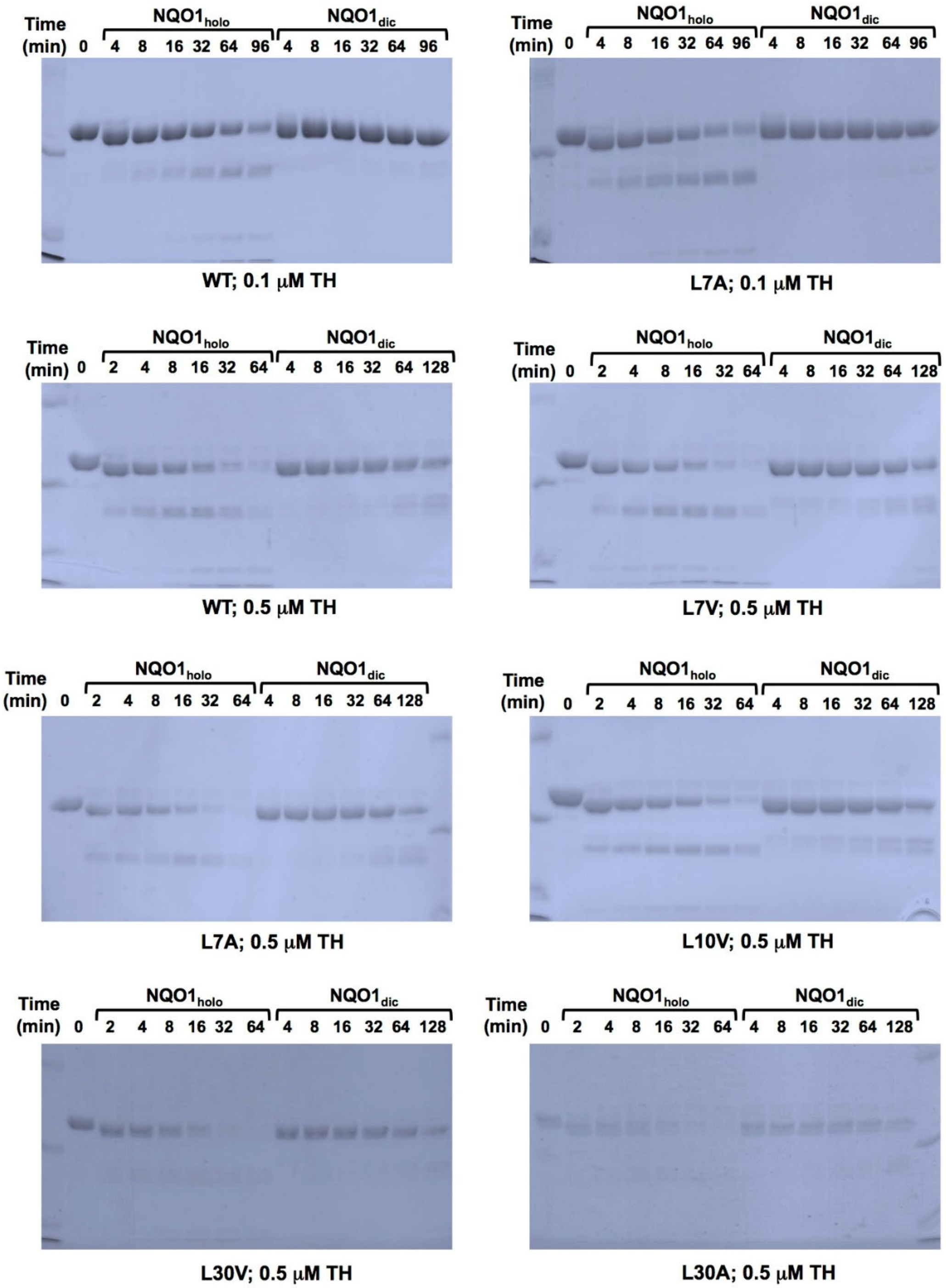

Figure S3. Representative SDS-PAGE analysis for the proteolytic kinetics of NQO1 variants with thermolysin. NQO1 samples were prepared at 10 μM NQO1 protein concentration in the presence of 100 μM FAD ($NQO1_{holo}$) or 100 μM FAD + 100 μM Dic ($NQO1_{dic}$). Thermolysin concentration is indicated for each experiment.

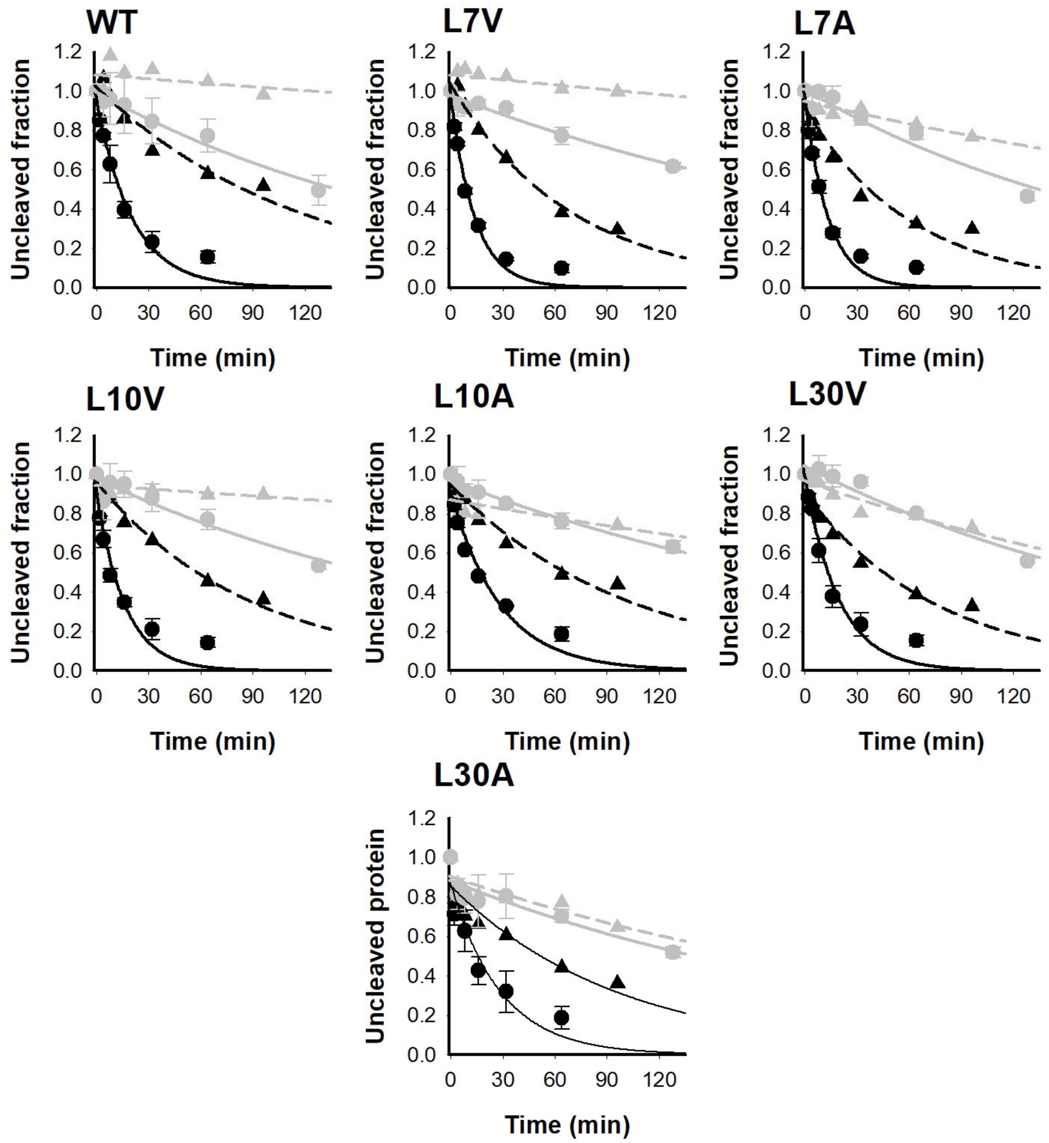

Figure S4. Proteolysis kinetics of NQO1 variants with thermolysin. $NQO1_{holo}$ is shown as black symbols and $NQO1_{dic}$ as grey symbols. Circles indicate a 0.5 µM thermolysin concentration (mean±s.d. from three experiments) and triangles show a 0.1 µM thermolysin concentration (from a single experiment). Lines are fittings using an exponential function.

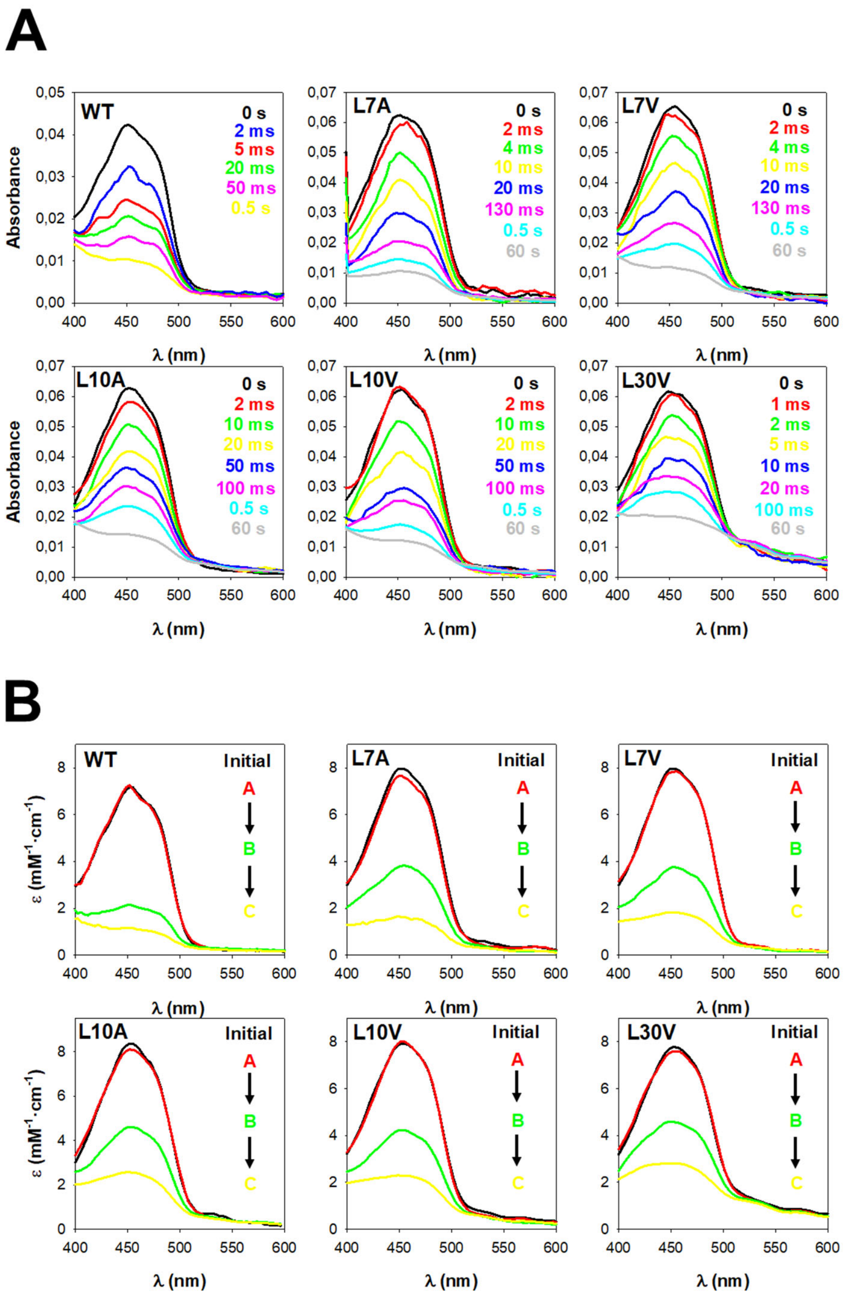

Figure S5. Reductive half-reaction of FAD bound to NQO1 variants with NADH. A) Spectral evolution in a 0-60 s timescale after mixing holo-NQO1 (7.5 μM) with NADH (7.5 μM) in 20 mM HEPES-KOH, pH 7.4, at 6 °C. Different colored lines correspond to the spectra at different reaction times. B) Spectral deconvolution of different species observed during the reaction when fitting to a three-state model. Different colored lines correspond to the different species along the reaction.

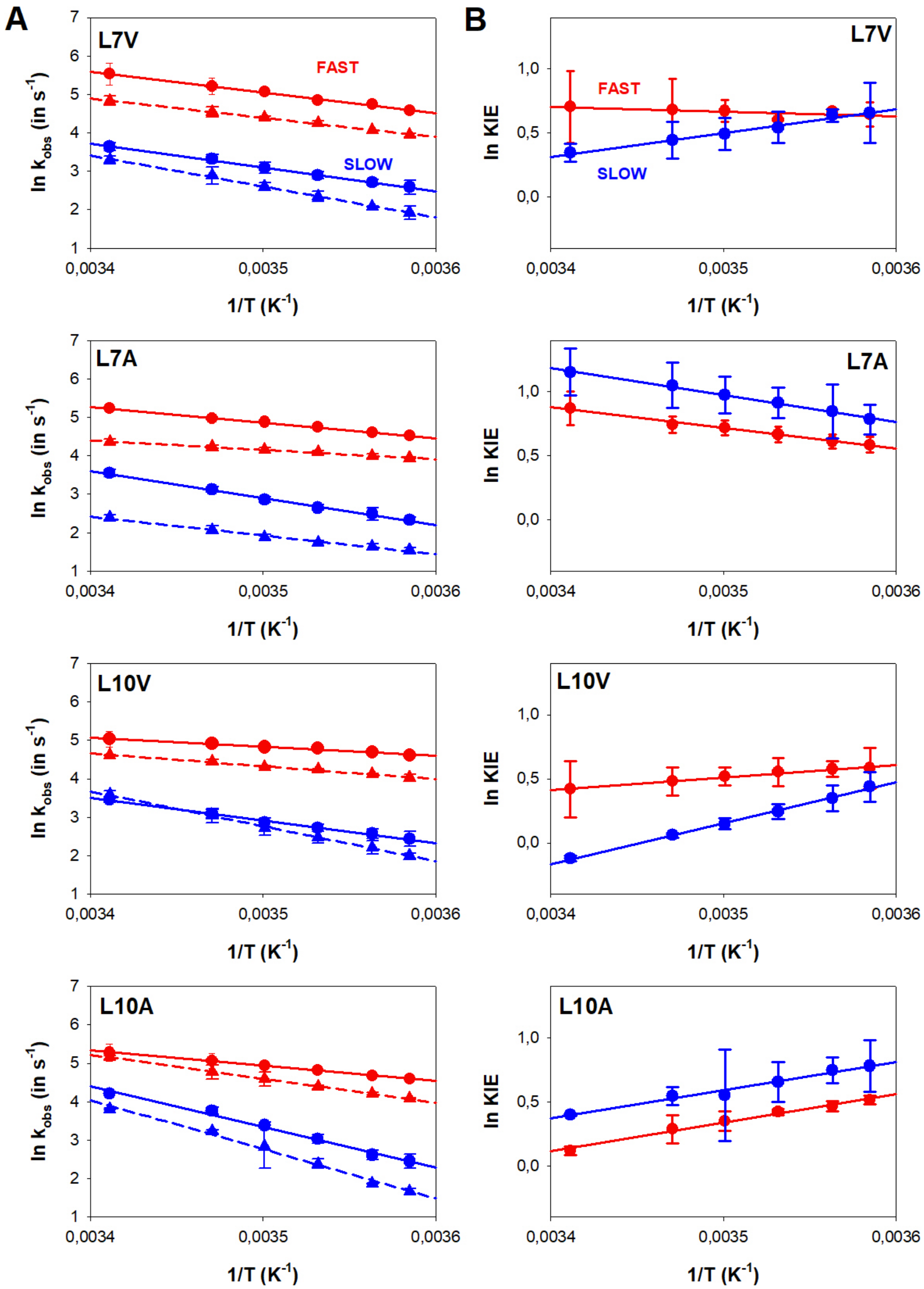

Figure S6. Temperature dependence of kinetic parameters for the two hydride/deuteride transfer (HT/DT) processes from NADH to NQO1. (A) Arrhenius plots of kinetic constants. Data for HT and DT are shown as circles and triangles, respectively. (B) Temperature dependence of the kinetic isotope effects (KIEs).

Table S1. Observed rate constants (k_{obs}) for partial proteolysis of NQO1 variants with thermolysin.

Variant	Species	Thermolysin concentration (μM)	k_{obs} (min^{-1})
**WT**	Holo	0.1	$8.4 \pm 1.2 \cdot 10^{-3}$
	Holo	0.5	$4.8 \pm 0.6 \cdot 10^{-2}$
	Holo+Dic	0.1	$6.4 \pm 8.0\ 10^{-4}$
	Holo+Dic	0.5	$5.0 \pm 0.4 \cdot 10^{-3}$
**L7V**	Holo	0.1	$1.4 \pm 0.1 \cdot 10^{-2}$
	Holo	0.5	$7.2 \pm 0.8 \cdot 10^{-2}$
	Holo+Dic	0.1	$8.1 \pm 4.8 \cdot 10^{-4}$
	Holo+Dic	0.5	$3.5 \pm 0.4 \cdot 10^{-3}$
**L7A**	Holo	0.1	$1.6 \pm 0.3 \cdot 10^{-2}$
	Holo	0.5	$7.4 \pm 0.9 \cdot 10^{-2}$
	Holo+Dic	0.1	$2.1 \pm 0.5 \cdot 10^{-3}$
	Holo+Dic	0.5	$5.2 \pm 0.8 \cdot 10^{-3}$
**L10V**	Holo	0.1	$1.1 \pm 0.1 \cdot 10^{-2}$
	Holo	0.5	$6.1 \pm 1.2 \cdot 10^{-2}$
	Holo+Dic	0.1	$6.5 \pm 3.9 \cdot 10^{-4}$
	Holo+Dic	0.5	$4.3 \pm 0.7 \cdot 10^{-3}$
**L10A**	Holo	0.1	$9.6 \pm 1.1 \cdot 10^{-3}$
	Holo	0.5	$3.5 \pm 0.6 \cdot 10^{-2}$
	Holo+Dic	0.1	$1.8 \pm 1.1 \cdot 10^{-3}$
	Holo+Dic	0.5	$3.5 \pm 0.3 \cdot 10^{-3}$
**L30V**	Holo	0.1	$1.3 \pm 0.2 \cdot 10^{-2}$
	Holo	0.5	$5.1 \pm 0.7 \cdot 10^{-2}$
	Holo+Dic	0.1	$3.3 \pm 0.5 \cdot 10^{-3}$
	Holo+Dic	0.5	$4.4 \pm 0.5 \cdot 10^{-3}$
**L30A**	Holo	0.1	$1.0 \pm 0.2 \cdot 10^{-2}$
	Holo	0.5	$7.4 \pm 1.0 \cdot 10^{-2}$

	Holo+Dic	0.1	3.3± 0.9· 10^{-3}
	Holo+Dic	0.5	4.1± 1.0· 10^{-3}

Table S2. Arrhenius parameters and KIEs for the HT/DT in the reduction of NQO1 variants by NADH/NADD. All values correspond to data obtained with equimolecular concentrations of the reactants in the stopped-flow equipment. (n > 3, mean ± s.d.). Analysis were performed as previously described (Anoz-Carbonell et al., 2020)..

**Step**	**HT**			**DT**			**KIE** [a]	**ΔE_{aDT-HT} (kcal·mol^{-1})**	**A_{HT}/A_{DT}**
	**k_{obsHT}** [a] **(s^{-1})**	**E_{aHT} (kcal·mol^{-1})**	**A_{HT} (s^{-1})**	**k_{obsDT}** [a] **(s^{-1})**	**E_{aDT} (kcal·mol^{-1})**	**A_{DT} (s^{-1})**			
**FAST**									
WT[b]	78±1	6.1±0.2	5.3±1.2·10^{6}	44±2	6.3±0.4	4.1±1.1·10^{6}	1.8±0.1	0.2±0.1	1.3±0.6
L7V	98±11	10.7±0.4	2.4±0.1·10^{10}	52±2	10.0±0.2	3.6±0.1·10^{9}	1.9±0.3	-0.7±0.1	6.7±0.5
L7A	92±8	8.0±0.2	2.0±0.1·10^{8}	51±1	4.8±0.1	3.3±0.1·10^{5}	1.8±0.2	-3.2±0.2	6.1±0.5·10^{2}
L10V	100±6	4.7±0.3	5.4±0.2·10^{5}	56±5	6.7 ±0.2	9.8±0.1·10^{6}	1.8±0.3	2±0.2	5.5±0.2·10^{2}
L10A	99±5	8.0±0.2	1.6±0.1·10^{8}	59±1	12.4 ±0.2	2.9±0.1·10^{11}	1.7±0.1	4.4±0.2	5.5±0.5·10^{4}
**SLOW**									
WT[b]	8.9±0.9	10.9±0.5	3.4±0.9·10^{9}	5.3±0.2	9.8±0.5	2.6±0.6·10^{8}	1.8±0.3	-1.1±0.2	13±6
L7V	13.2±2.4	12.3±0.4	5.9±0.2·10^{10}	6.9±1.2	16.0±0.4	2.2±0.1·10^{13}	1.9±0.6	3.7±0.2	2.6±0.2·10^{3}
L7A	10.3±0.8	14.0±0.5	8.0±0.3·10^{11}	4.7±0.3	9.5±0.5	2.0±0.1·10^{8}	2.2±0.3	-4.5±0.4	4.0±0.3·10^{3}
L10V	11.3±2.2	11.7±0.5	1.6±0.1·10^{10}	7.3±0.5	18.1±0.4	9.6±0.1·10^{14}	1.5±0.4	6.4±0.4	1.7±0.1·10^{2}
L10A	11.6±2.2	21.0±1.1	3.2±0.2·10^{17}	5.3±0.4	25.4±0.1	4.3±0.2·10^{20}	2.2±0.6	4.4±0.3	7.4±0.8·10^{4}

[a] Experiments carried out at 6ºC. [b] From (Anoz-Carbonell et al., 2020).

Supplementary references

1. Anoz-Carbonell, E., Timson, D. J., Pey, A. L., & Medina, M. (2020a). The catalytic cycle of the antioxidant and cancer-associated human NQO1 enzyme: Hydride transfer, conformational dynamics and functional cooperativity. Antioxidants, 9(9), 1–22. https://doi.org/10.3390/antiox9090772
